# Multiple Sclerosis: Roles of miRNA, lcnRNA, and circRNA and Their Implications in Cellular Pathways

**DOI:** 10.3390/ijms25042255

**Published:** 2024-02-13

**Authors:** Giovanni Luca Cipriano, Giovanni Schepici, Emanuela Mazzon, Ivan Anchesi

**Affiliations:** IRCCS Centro Neurolesi “Bonino-Pulejo”, Via Provinciale Palermo, Strada Statale 113, Contrada Casazza, 98124 Messina, Italy; giovanniluca.cipriano@irccsme.it (G.L.C.); giovanni.schepici@irccsme.it (G.S.); ivan.anchesi@irccsme.it (I.A.)

**Keywords:** multiple sclerosis, microRNAs, long non-coding RNAs, circular RNAs, biomarkers, therapeutic strategies

## Abstract

Multiple sclerosis (MS) is a degenerative condition characterized by axonal damage and demyelination induced by autoreactive immune cells that occur in the Central Nervous System (CNS). The interaction between epigenetic changes and genetic factors can be widely involved in the onset, development, and progression of the disease. Although numerous efforts were made to discover new therapies able to prevent and improve the course of MS, definitive curative treatments have not been found yet. However, in recent years, it has been reported that non-coding RNAs (ncRNAs), including microRNAs (miRNAs), long ncRNAs (lncRNAs), and circular RNAs (circRNAs), acting as gene expression regulators, could be used as potential therapeutic targets or biomarkers to diagnose and fight MS. In this review, we discussed the role of miRNAs, lncRNAs, and circRNAs, as well as their expression level changes and signaling pathways that are related to preclinical and human MS studies. Hence, the investigation of ncRNAs could be important to provide additional information regarding MS pathogenesis as well as promote the discovery of new therapeutic strategies or biomarkers.

## 1. Introduction

Multiple sclerosis (MS) is a chronic autoimmune and demyelinating disease that affects the central nervous system (CNS), leading to progressive neurodegeneration [1]. MS mainly affects young adults with a female predominance, aged between 20 and 40 years old [2].

Despite relapsing–remitting MS (RRMS) being the most common subtype, the disease includes primary progressive MS (PPMS), secondary progressive MS (SPMS), and progressive relapsing MS (PRMS) subtypes [3,4].

Usually, the clinical signs of the disease start with a relapsing-remitting phase characterized by unilateral optic neuritis, diplopia, and sensory deficits, which can stabilize after several days. Persisting signs of CNS dysfunction are able to lead to cognitive deficits and irreversible disabilities [2].

MS acute forms can be aggressive and may quickly lead to death, with aggressive cases occurring in childhood and pediatric age [5,6].

Although it is not fully clarified, it was largely confirmed that in MS, the involvement of autoreactive T cells, including T helper (Th-1), CD4+ T cells, and Th17, occurs through the cytokine’s secretion and inflammatory cascade activation, which subsequently leads to the formation of demyelinating plaques. Demyelination, as a consequence, induces both microglia activation and oxidative stress, triggering the neurodegenerative processes that promote axonal damage and neuronal death [7].

The balance between the complex genetic components and epigenetic regulation may be crucial to the susceptibility, onset, and progression of MS [1].

Epigenetic marks can be inherited throughout life by modulating gene expression, which can determine the MS phenotype without inducing changes in the DNA sequence [8].

In this regard, epigenetic alterations, including DNA methylation, post-translational histone modifications, and non-coding RNA (ncRNA) regulation, play an important role in the onset and progression of MS, as well as in myelination and remyelination processes [8,9,10].

Due to their ability to modulate genes’ expression and their role in physiology and various pathological processes, ncRNAs, including microRNAs (miRNAs), long ncRNAs (lncRNAs), and circular RNAs (circRNAs), could be considered epigenetic factors involved in immune regulation and MS pathogenesis [11].

miRNAs are widely expressed in the CNS and in the immune system. They are involved in the regulation of target gene expression in a wide range of biological processes, including cell differentiation, proliferation, hematopoiesis, organogenesis, and apoptosis [12,13,14].

It was estimated that miRNAs target about 33% of human genes, and with them, they constitute a network able to regulate physiological and pathological processes [15].

miRNA biogenesis begins in the nucleus, where RNA polymerase II produces the primary transcripts (pri)-miRNA. A further process of pri-miRNAs performed by the enzymes Drosha and DGCR8 leads to the production of one or more precursor (pre)-miRNAs. The pre-miRNAs are translocated by Exportin-5 (XPO5) from the nucleus to the cytoplasm, where they are dissociated by Dicer into potentially mature miRNA duplexes. Now, only one filament is incorporated into the RNA-induced silencing complex (RISC), which consists of Dicer and TAR-RNA binding protein (TRBP), while the other strand is degraded. The guide strand remaining binds to the 3′-untranslated region of its target mRNA to induce or inhibit the translational repression of the target protein [16,17].

Although they were initially considered spurious transcriptional noise, the advent of Next Generation Sequencing (NGS) revealed an abundance of lncRNAs in the human genome, as well as their involvement in several biological processes [18,19].

LncRNAs are able to regulate every step of gene expression, affecting transcriptional, post-transcriptional, and translational processes, acting as miRNA sponges, modulating apoptosis, intracellular trafficking, and molecular scaffolding [18,20,21,22].

Despite the precise origins yet to be defined [18], and unlike miRNAs, lncRNA biogenesis can occur both in the nucleus and cytoplasm [23].

Improperly defined as a group of RNAs that do not translate into proteins, lncRNAs are transcribed by Pol II or Pol III [24].

According to their genomic organization as well as their relation to protein-coding genes, lncRNAs can be classified as antisense lncRNAs, intronic lncRNAs, intergenic lncRNAs, sense lncRNAs, enhancer RNAs, and circular RNAs. The latter represents a class of lncRNAs with a covalently closed continuous loop lacking 5′–3′ polarity [24,25].

Although most of them were found in the cytoplasm, circRNAs were also identified in the human cell’s nucleus. They are classified as exon-intron circRNAs (EIciRNAs), circular intron RNAs (ciRNAs), and exon circRNAs (ecircRNAs) [26,27].

Functionally, their intrinsic circular structure makes circRNAs most resistant to RNAse degradation compared to linear-structure ncRNAs [28].

Also, circRNAs can act as miRNA sponges as well as modulators of several biological processes, including cell proliferation, apoptosis, and cell differentiation [29,30]. Notably, ciRNAs are widely expressed in brain tissue, where they are involved in neural development [31].

Based on this, it could be interesting to evaluate and investigate the role of ncRNAs, including miRNAs, lncRNAs, and circRNAs, as well as their alterations in CNS damage. The purpose of this study was to summarize preclinical and human studies that examined ncRNAs and to evaluate their levels, effects, and signaling pathways that are connected to MS.

## 2. Methodology

To write this narrative review, we carried out a Pubmed search considering these inclusion and exclusion criteria: articles published between 2020–2023, excluding the preceding years, that had as topic non-coding RNAs (lncRNAs, miRNAs, and circRNAs) in the context of multiple sclerosis were included in our research, excluding other topics deviating from the specific aim of the work. We searched for the following keywords: “epigenetic regulation and multiple sclerosis” (126 papers), “Epigenetic and non-coding RNA and multiple sclerosis” (540 papers), “epigenetic AND miRNA AND multiple sclerosis” (29 papers), “epigenetic AND long non-coding RNA AND multiple sclerosis” (5 papers), “epigenetic AND circRNA AND multiple sclerosis” (3 papers), “(multiple sclerosis[Title/Abstract]) AND (miRNA[Title/Abstract])” (151 papers), and “(multiple sclerosis[Title/Abstract]) AND (circRNA[Title/Abstract])” (14 papers).

## 3. The Role of Non-Coding RNAs in Multiple Sclerosis

MS is the most prevalent inflammatory and chronic disease of the CNS, affecting more than 2 million people worldwide, with patients experiencing cognitive deficits, impaired mobility, and loss of sphincter control [4,32].

The progression of MS leads to more diffuse inflammation and neurodegenerative processes [33,34], which are driven by macrophage and T-lymphocyte infiltrations that push forward to the myelin sheath’s destruction [35,36].

Despite the absence of curative treatments able to stop the destruction of the nervous tissue, nowadays, MS therapies are mainly based on immunomodulatory and anti-inflammatory drugs [37,38].

Consequently, one of the challenges today, given the lack of specific and valid diagnostic and prognostic biomarkers, is the development of effective MS therapies. Since their involvement in several biological and physiological processes, including immunity and inflammation, in recent years, ncRNAs, including miRNAs, lncRNAs, and circRNAs, have been investigated as potential molecular approaches for early diagnosis and MS treatment [11].

CNS injuries that occur during MS pathogenesis are closely related to miRNA dysregulation, influencing activation, differentiation, and the function of T and B cells, thus contributing to the progression and development of the disease [39].

Furthermore, it was discovered that some miRNAs are able to promote pro-inflammatory T cell activation and inhibit their regulatory functions, favoring an imbalance of the immune response. It is noteworthy that miR-155 can modulate the regulatory T-reg function and increase Th1 and Th17 responses. Hence, this imbalance in T cells can trigger a higher pro-inflammatory response and MS progression [40,41].

When upregulated, miRNAs can act as negative regulators of Th17/Th1 cell differentiation in MS patients. In this regard, it was found that miR-326 was overexpressed in Th17 cells that produce IL-17 in RRMS patients, and it promotes Th17 cell differentiation by inhibiting Ets-1 [42].

Notably, miR-590 was overexpressed in relapse cases of MS, leading to Th17 cell differentiation through Tob1, a member of the anti-proliferative (APRO) family of proteins involved in the progression and control of the cell cycle. Consequently, miR-590 overexpression led to Th17 cell pathogenicity, promoting an increase in pro-inflammatory molecules including interleukin (IL)-23R, CXCL3, and CSF2 [43].

Also, an increase in miR-27a was found in purified CD4+ T cells of MS patients in the relapsing phase to promote the differentiation of T naïve cells to Th17 cells, probably influencing the transforming growth factor beta (TGF-β) signaling pathway [44].

MiR-448 was significantly increased in MS patients, inducing a Th17 response through the protein tyrosine phosphatase non-receptor type 2 (PTPN2) [45].

In the same way, miRNA let-7e was significantly upregulated in the experimental autoimmune encephalomyelitis (EAE) mouse model, according to a mechanism that targeted IL-10 and enhanced the Th1 and Th17 cell ratios [46].

Like the findings in T cells, the presence of dysregulated miRNAs such as miR-326 and miR-155 [42] in B cells can also promote the differentiation of B cells into antibody-producing plasma cells, which are able to target the CNS. In this regard, the blood-brain barrier (BBB) disruption driven by both an upregulation of matrix metallopeptidase-9 (MMP-9) and a downregulation of miR-320a contributes to the exacerbation of the pathology [47]. Overall, the effects mediated by miRNAs can contribute to the dysregulation of the immune response shown in MS patients [48,49,50].

Moreover, it was observed that both the miR-106b-25 cluster and the miR-17-92 cluster were deregulated in the B cells of RRMS patients treated with immunomodulatory therapy. The analysis of miRNA-mRNA interaction demonstrated that signaling pathways including B cell receptor, phosphatidyl-inositol-3-kinase (PI3K), and phosphatase and tensin homology (PTEN) were the most affected in MS patients [51], with also an enhancement of cytokines production, such as lymphotoxin and tumor necrosis factor α (TNF-α), due to an overexpression of miR-132 [52].

Moreover, miRNAs can also regulate the immune function of dendritic cells and macrophages, both involved in MS pathogenesis [53].

miR-146a is able to modulate dendritic cell activation, influencing their ability to regulate immune responses, thus promoting the dysregulated immune response characteristic of MS patients, while upregulated miR-155 was found in peripheral circulating monocytes of RRMS patients. which was found to induce the M1polarization state of human macrophages and microglia, thereby forcing pro-inflammatory responses [54,55].

On the other hand, miR-124 modulates resident microglia cells in the CNS, as well as macrophage activation and monocytes. It was noted that miR-124 downregulation is able to act toward M2 phenotype differentiation thanks to TNF-α downregulation and an upregulation of TGF-β, arginase-1, and FIZZ1, hence making it an important target to establish an anti-inflammatory M2 phenotype in MS patients [56].

Increasing evidence supports the idea that lncRNAs can also be involved in MS pathogenesis [11].

LncRNAs are a class of ncRNAs with lengths of more than 200 nucleotides that act through different pathways that can be involved in several functions, including chromatin remodeling, miRNA sponging, and regulation of the protein’s stability. Furthermore, it was reported that lncRNAs can contribute to different neuronal biological processes such as CNS development, cell differentiation, myelination, neurotransmission, and neuroinflammation [11,57].

Recently, it was suggested that dysregulated lncRNAs were involved in MS pathogenesis, leading to an increase in the pro-inflammatory response induced by microglia polarization versus a M1 phenotype rather than a low M2 phenotype [58].

In this regard, the lncRNA growth arrest-specific transcript 5 (GAS5) was found to be upregulated in the microglia of MS patients. It suppressed the transcription of TRF4, a key factor able to inhibit M2 polarization, in the EAE mouse model [59].

Also, the lncRNA metastasis-associated lung adenocarcinoma transcript 1 (MALAT1) was involved in MS. It was found that the expression of MALAT1 was decreased in the spinal cords of EAE mice and in primary macrophages as well as stimulated splenocytes. MALAT1 downregulation by specific siRNAs led to the polarization of macrophages towards the M1 inflammatory phenotype. It was also reported that MALAT1 downregulation may influence MS course through the modulation of pro-inflammatory Th1 and Th17 cells, leading to a decrease in T-regs differentiation as well as an increase in Th1 and Th17 inflammatory phenotypes [60].

Additionally, there was an increase in the expression of P21-associated ncRNA DNA damage activated (PANDA), taurine-up-regulated gene 1 (TUG1), and nuclear paraspeckle assembly transcript 1 (NEAT1), three lncRNAs involved in immune responses and DNA damage responses in MS patients [61].

PANDA is a lncRNA involved in DNA damage related to the p53 pathway [61]. It can modulate the cell cycle by suppressing genes related to the apoptotic process, and its dysregulation in neurons and oligodendrocytes leads to both free radical production and apoptosis [61,62]. TUG1 is involved in DNA damage and is involved in cell cycle regulation and apoptosis through p53 modulation [63]. Its upregulation in peripheral blood mononuclear cells (PBMCs) and serum of RRMS patients was reported, along with its ability to target miR-20a-5p, which leads to the activation of p38 MAPK as well as the production of inflammatory cytokines [61,64,65].

The upregulation of NEAT1 in MS patients leads to an increase in the pro-inflammatory cytokine IL-8 through the modulation of splicing factor proline/glutamine-rich (SFPQ) [66].

Another lncRNA, UCA1, is involved in the cell cycle and proliferation of cancer cells [67]. Its upregulation in the blood of MS patients leads to a block of cell cycle inhibitors like p27, increasing T cell proliferation [68].

The lncRNA, linc-MAF-4, was significantly upregulated in MS patients’ PBMCs. Linc-MAF-4 is able to act on the Th1/Th2 cell ratio, promoting pro-inflammatory processes and targeting MAF, a transcription factor of Th2 cells.

Along with linc-MAF-4, lncDDIT4 was also upregulated in MS patients. It is involved in the modulation of inflammatory and immune responses. In the presence of DNA damage, DDIT4, a cytoplasmic protein, was upregulated, inhibiting the mTORC1 pathway and thus regulating the immune response through Th17 differentiation and IL-17 production. Hence, the lncDDIT4 direct binding of DDIT4 leads to its overexpression and suppression of Th17 differentiation [69,70].

circRNAs belong to a new class of ncRNAs that play an important role in the complex network of gene regulation.

After their synthesis, they are able to exert many peculiar functions, such as gene expression regulation and influencing transcription by acting as miRNA sponges, and some of them are even translated into proteins. circRNAs, with their stability given by the closed circular structure and their tissue specificity, are emerging as feasible biomarkers and therapeutic targets, significantly enriching the understanding of MS pathogenesis [71,72].

One important piece of evidence regarding circRNAs is the changes in the expression levels of Gasdermin B (GSDMB), a gene frequently related to asthma susceptibility and autoimmune disorders.

GSDMB alternative splicing (AS) leads to the rise of an ecircRNA, also known as hsa_circ_0106803, which was found to be highly expressed in several regions of the brain, resulting in upregulation, as reported in RRMS patients’ PBMCs. Alongside these data, comparative analysis performed on circRNA expression profiling of peripheral blood leucocytes from MS patients reported a downregulation of circ_0005402 and circ_0035560, both located in chromosome 15 inside the ANXA2 gene, in these patients [73,74,75].

In confirmation of the involvement of GSDMB in MS, it was shown that the reduction of GSDMB in memory CD4+ T cells led to an increase in cytokine production, including TNF, IL-13, and IL-16 [76].

Additionally, it was reported that a GSDMB D6 isoform led to an alteration in the expression levels of TGF-β1 and MMP9 [77]. Indeed, a TGF-β1 reduction in MS patients’ leukocytes On the contrary, an increase in TGF-β1 expression levels was found in the serum of MS patients treated with immunomodulatory molecules [78,79].

GSDMD activation was also linked to inflammasome-signaling-dependent pyroptosis, thus promoting the inflammatory process associated with MS [80,81].

Therefore, the stability of these circRNAs, as well as their presence in peripheral whole blood, could suggest GSDMD circRNA as a potential MS biomarker [82].

Another interesting circRNA is circ_HECW2. In an experimental MS study, its increment led to endothelial-mesenchymal transition (EndoMT), a process that contributes to BBB dysfunction. circ_HECW2 was also involved in neural growth regulation in human brain microvascular endothelial cells (HBMECs) induced by lipopolysaccharide (LPS), according to a mechanism where circ_HECW2 modulates neuronal growth regulator 1 (NEGR1) expression through miR-30e-5p sponging. Its silencing also led to suppression of apoptosis and endoMT, as well as favoring cell proliferation in LPS-induced HBMECs [83].

In the same way, dysregulation of circ_0043813, derived from the signal transducer and activator of transcription 3 (STAT3) gene, is involved in Th17 differentiation and inflammatory processes related to MS patients [84].

Overall, evaluating the expression levels of ncRNAs, as well as verifying their involvement and mechanisms underlying the MS pathogenesis, could be useful in finding new diagnostic and predictive tools for the disease.

## 4. miRNAs in Multiple Sclerosis

### 4.1. Pathway—miRNAs

MiRNAs are not just possible future markers that could be implemented in the clinical practice of MS. Their involvement in biological processes and signaling pathways is crucial to better understanding the molecular landscape underlying the complex MS pathophysiological mechanisms. 

The pilot study by Sağir and colleagues highlighted the role of specific microRNAs, such as miR-132-3p, miR-106b-5p, and miR-19b-3p, in MS patients, notably examining their expression and regulation with BDNF production and the subsequent clinical outcome. They showed a negative correlation with BDNF serum levels in SPMS and a possible compensatory mechanism in RRMS, where, along with increased disability as indicated by higher EDSS scores, they found a correlation between elevated BDNF levels and decreased levels of the studied miRNAs. These findings are in accordance with the literature regarding the role of these miRNAs in inflammation and neurodegenerative pathways, confirming the interaction with this important neurotrophin [85].

miRNAs are able to influence the role and action of different proteins, leading to different scenarios of pathology in MS. For instance, the expression of neurotrophins, heat shock proteins, and SIRT1 is impaired in multiple sclerosis. A decrement in the aforementioned neurotrophic factors and a simultaneous associated increment in the expression of miR-132-3p, miR-34a, and miR-132 in PBMCs may be an indicator of inhibition of the neuroprotective function of these cells. These events may be associated with the transition of the immune system towards inflammation, which leads to the subsequent production of IFN-γ and IL-17A in T cells, responsible for the differentiation and activation of Th1 and Th17, maintaining the chronic inflammation that can be seen in MS. These statements show that the levels of neurotrophins, heat shock proteins, and peculiar mRNAs might be taken into account as future biomarkers of neurodegenerative processes and CNS inflammation in the context of MS patients [86]. Also, high levels of expression of miRNA-21, miRNA-155, and miRNA-182 have been detected in the cerebrospinal fluid of MS patients compared to controls, showing an important correlation with the levels of inflammatory cytokines such as IL-1β, IL-6, TNF-α, and hs-CRP levels in CSF, suggesting their involvement in the inflammatory processes of MS [87]. Other important evidence shows how the manipulation of miRNA expression, such as the KO, sheds light on their role in MS lesion progression and onset. It was found that the knock-out of miRNA-146a leads to higher levels of IL-1β, TNF, IL-6, IL-10, CCL3, and CCL2 compared to WT mice. MiR-146a was found to be upregulated in all types of MS brain lesions, with the highest expression observed in active lesions, highlighting its pivotal role in MS lesions and their progression, acting as a key regulator not only of microglia inflammatory cytokine expression but also on the entire MS pathophysiology [88]. Interestingly, the expression patterns of Drosha, Pasha (DGCR8), and Dicer genes were assessed along with the major pro-inflammatory and anti-inflammatory cytokines such as IFN-α, IFN-β, and IL-6. The highest transcription activity of Drosha was observed in RRMS patients, while the ratio of expression was down-regulated in male and female patients with SPMS. Considering the studied cytokines, the increase in the expression ratio of IL-6 in SPMS patients and the decrease in transcript abundance of INF-α and INF-β cytokines are consistent with the progression of the disease [89]. The pro-inflammatory microRNA-155 (miR-155) was shown to be strongly upregulated in the serum and in the CNS lesions of MS patients. The total knockout of miR-155 in the EAE mouse model resulted in resistance from infiltrating Th17 T cells. The total miR-155 KO time revealed reductions in T cells, macrophages, and dendritic cells when compared with wild-type controls. Furthermore, the researchers have shown a difference in the role of miR-155, depending on the analyzed cell types. Deletion of miR-155 in T cells shows a reduction in the disease severity; deletion of miR-155 in dendritic cells also resulted in a significant reduction in the disease development, while miR-155 deletion in macrophages has not shown an interesting impact on the disease severity. These evidences result in a more concrete understanding that even a single microRNA is able to induce different responses depending on the cell type in which it is expressed [90].

Another interesting step forward to a broader understanding of the immune component of MS is linked to Th17 differentiation. MiRNA-467b has been shown to directly target eIF4E, which, in turn, is a crucial component of Th17 differentiation. This precise miRNA could be useful against multiple sclerosis treatment thanks to its capacity to delay the disease progression, alter the process of Th17 differentiation, and subsequent worsening of the patient [91]. microRNA, named miR-485, was found to play a pivotal role in Th17-mediated responses involved in MS. Its overexpression has been linked to a decrease in the cytokine response, mitigating the effect of EAE in mice and also inducing STAT3 downregulation and the subsequent effect on the Th17 cell population. Moreover, attention has been focused on the role that miRNA let-7 exerts, negatively regulating Th17 cells. It was found that it is able to directly target the cytokine receptors Il1r1 and Il23r as well as the chemokine receptors Ccr2 and Ccr5, becoming an interesting possible indicator of Th17 immune modulation in MS pathology [92,93].

Delving into the role of immune modulation in Th17 cells may further help to better understand MS pathophysiology. The role of miR-21, miR-146a, miR-155-3p, miR-155-5p, and miR-301a has been highlighted in this study, where the upregulation of the latter is linked with their interaction with HSP-70 and the subsequent modulation of the Th17 response, presenting a potential mechanism for modulating the autoimmune response and the progression of MS [94].

Since one of the hallmarks of MS is indeed the demyelinating process, attention has also been brought to the role that miRNAs exert in this context. Important findings are related to the possible therapeutic role of mir-145-5p. It has been reported that it is well expressed in oligodendrocyte progenitor cells but downregulated during their differentiation, thanks to the concurrent prevention of the expression of the myelin gene regulatory factor (MYRF) into oligodendrocytes. It was also demonstrated that miRNA-145-5p knockdown leads to spontaneous differentiation of OPCs, suggesting its therapeutic implications in demyelinating diseases, such as multiple sclerosis, where remyelination is a critical aspect of disease management [95]. In addition to this, the attention of the research has also been focused on the molecular landscape of gray matter lesions, which are related to the loss of matter that involves MS pathophysiology. where several miRNAs have been identified to be both upregulated and downregulated in this clinical context. Thanks to bioinformatics analysis, they were linked to molecular pathways associated with axonal guidance, TGF-β signaling, and FOXO signaling that significantly indicate their involvement in critical biological processes relevant to MS pathology [96]. Another important study highlights the change in miRNA expression and profile, which are differentially regulated in oligodendrocytes during the initiation and progression of EAE. In this MS model, fifteen and three miRNAs were upregulated and downregulated as well, respectively. Investigating their transcriptional profile during the disease model peak also showed activation in the peroxisome, FoxO signaling, glutathione metabolism, and ferroptosis in KEGG [97]. Other important evidence regarding white matter lesions in MS pathology involves the role of miRNAs and their interaction with the synaptic protein Syt7. It has been shown that the upregulation of five microRNAs, such as miR-330-3p, miR-4286, miR-4488, let-7e-5p, and miR-432-5p, which commonly share synaptotagmin-7 as a molecular target, indeed influences protein transportation toward the synapses and also induces its accumulation in the soma, leading to impaired neuronal stability and function [98].

The research also intriguingly shifted its focus to the impact of vitamin D metabolism on MS. Notably, it highlighted compelling evidence regarding the role of miR-125a-5p in influencing the metabolism of vitamin D, specifically its contribution to the downregulation of the vitamin D receptor. The upregulation of the cited miRNA is involved directly or indirectly in VDR, since the latter and miR-125a-5p have been found to be co-localized in the same neurons. Also, the subsequent molecular inhibition of the miRNA led to a decrease in the VDRs. Thus, miR-125a-5p may be taken into consideration as a potential target to intervene in this important metabolism, acting very closely on the receptor itself. To further confirm the interesting role that these ncRNAs play in vitamin D metabolism, miRNA-22 has also been associated with vitamin D metabolism and levels. What was found is that miRNA-22 levels were significantly downregulated in MS patients in comparison to controls, also showing lower VD and VDR levels in comparison to controls [99,100].

**Figure 1:**
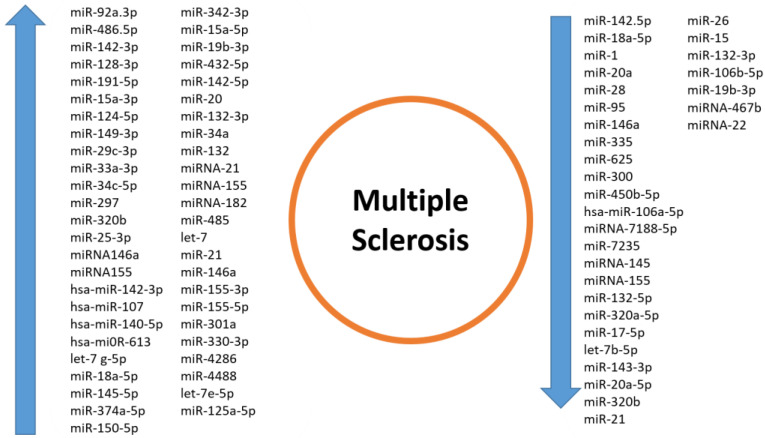
This figure represents the upregulated/downregulated miRNAs in multiple sclerosis.

Interestingly, miR-320b, miRNA-155, miR-18a-5p, miR-132-3p, miR-19b-3p, and miR-146a have shown different alterations in the level of expression, and they are involved in peculiar and specific pathways with both up and down regulation, depending on the state of the experiments and possibly the plethora of clinical and molecular differences underlying the complex landscape of MS pathophysiology (Table 1 and Figure 1).

### 4.2. miRNAs as MS Biomarkers

Researchers have increasingly focused on microRNAs (miRNAs) in the context of neurodegenerative diseases such as multiple sclerosis (MS) due to their critical roles in gene expression regulation and their involvement in various biological processes. MiRNAs are small, non-coding RNAs that are able to modulate processes like immune function, inflammation, and many other biological activities, which are key aspects of MS pathophysiology. The potential of miRNAs serving as biomarkers for MS diagnosis, prognosis, and treatment response, together with their potential as therapeutic targets, has made them significantly important in MS research. Their different expression in MS patients compared to healthy controls and their occurrence in readily accessible biological fluids like blood and cerebrospinal fluid further enhance their importance in studying this complex autoimmune disorder.

In this study carried out by Geiger et al., the peculiar association between circulating microRNAs and structural and functional MRI parameters was investigated in MS patients. Their key results focused on the attention of four miRNAs that were associated with different parameters to use in the detection and prognosis of MS patients. In particular, they studied the differentiation of MS subtypes by miR-143.3p, miR-92a.3p, and miR-486.5p for white matter lesion volumes and the correlation of miR-142.5p with both creatinine concentrations and functional connectivity. The detection of serum miR-143.3p levels to distinguish between MS subtypes places emphasis on the potential it possesses as a biomarker for disease classification and monitoring. In addition, increased levels of miR-92a.3p and miR-486.5p were correlated with greater total white matter lesion volumes in the cervical spine, suggesting a possible function of these miRNAs linked to the process of structural damage in MS. Moreover, the decreased levels of miR-142.5p showed a peculiar correlation pattern, in which they were correlated with reduced total creatinine concentrations, indicating a metabolic connection between these two data. Moreover, the positive association of miR-142.5p with functional connectivity strength between the retrosplenial cortex and temporal pole and the negative association of miR-92a.3p and miR-486.5p with connectivity strength between the lateral temporal cortex and posterior inferior parietal lobule provide direct insights, supplemented by the MRI functional analysis, into the impact of how these miRNAs affect the brain network functionality in MS [101].

Another interesting marker identified in the CSF of RRMS is miR-142-3p. Its overexpression in patients has been interestingly linked to an increased level of IL-1β and its signaling, increasing neuronal excitability, which is potentially a new indicator of disease progression. Its biological role in the pathology positively correlates with disease progression, and it is associated with increased levels of signaling as well as increased neuronal excitability [102].

The role of serum detectable miRNAs has also been investigated in a four-year follow-up study, where the main actors identified were miR-191-5p, miR-128-3p, miR-24-3p, and miR-223-3p. MiR-191-5p was identified as a significant biomarker, overexpressed in RRMS compared to the controls. Its temporal changes, aligned with MRI activity, suggest a role in the neurodegenerative processes underlying MS. The upregulation of miR-191-5p in RRMS implies its involvement in inflammatory cascades and neurodegenerative processes, notably during the peak of the disease symptoms. miR-128-3p shows a distinct and peculiar pattern, resulting in a mainly elevated PPMS with a stabilization in its own expression levels over time. These data, in association with PPMS, indicate miR-128-3p’s potential role in chronic neurodegeneration, likely influencing the mechanisms that drive progressive disability independently from acute inflammatory activity. Conversely, miR-24-3p and miR-223-3p have not been linked with MS subtype-specific differences. Nevertheless, the negative correlation of miR-223-3p with T1 lesion volumes in SPMS and PPMS and its temporal variability linked to the disease periods of relapsing suggest its involvement in more dynamic aspects of the disease itself. Further, miR-223-3p could be implemented as a potential marker for disease progression and response to treatment in relation to relapse dynamics. The temporal variability of these miRNAs, along with MRI scans and clinical markers, gives strength to the possibility of clinical implementation and practical usage of these non-coding RNAs in MS pathophysiology [103].

The differential expression of miRNAs in various biological compartments and fluids, such as CSF, serum, and PBMCs, is actually essential for the discovery of new biomarkers that could help us with MS activity and progression. An interesting paper sheds light on a novel set of miRNAs that emerged as novel upregulated markers of disease and intrathecal inflammation, such as miR-15a-3p/124-5p/149-3p/29c-3p/33a-3p/34c-5p/297, confirming miRNAs contribute to MS inflammatory and neurodegenerative aspects [104].

Another step forward for a better understanding of miRNAs role as early or late biomarkers in MS has been highlighted thanks to this study in which miR-320b and miR-25-3p upregulation have been correlated with multiple sclerosis severity. As a matter of fact, higher levels of miR-320b in the early stages of MS are correlated with a more benign development of the disease, suggesting a protective role for the latter. On the other hand, miR-25-3p baseline levels tend to be associated with benign MS at year 10, which supports the role of miRNAs as early biomarkers of long-term outcomes [105].

In this work, the focus is on the peculiar roles of miRNA146a and miRNA155. It was demonstrated how these two miRNA expression levels are related to MS pathogenesis and to the disability status of patients, becoming two new interesting biomarkers to take into account for future analysis related to the complex landscape of MS molecular regulation [106].

In this bioinformatics analysis, four main miRNAs have been investigated for their role in MS, highlighting a whole complex protein–protein interaction. Several hub genes have been identified, including JUN, FPR2, AKT1, POLR2L, LYZ, CXCL8, HBB, CST3, CTSZ, and MMP9, especially LYZ and CXCL8. The researchers built a miRNA-mRNA regulatory network and found that hsa-miR-142-3p, hsa-miR-107, hsa-miR-140-5p, and hsa-mi0R-613 were the most important expressed and upregulated miRNAs. In this peculiar context, they are thought to be implicated in the regulatory process underlying MS pathogenesis [107].

Another microRNA that was shown to be involved in the pathogenesis of MS is miR-18a-5p. Its downregulation in MS contributes both to the regulation of Th1 cells, which are implicated in MS immune control since they secrete gamma interferon, which, in turn, exacerbates inflammatory symptoms of the pathology, and to the differentiation of Th17 cells [108].

Interestingly, MS has been studied in women to better understand how pregnancy behavior affects the pathology symptoms. It was found that miR-1, miR-20a, miR-28, miR-95, miR-146a, miR-335, and miR-625 downregulation ameliorate the clinical symptoms of pregnant MS patients. These miRNAs have been predicted to target important inflammation-regulating genes. Among these, we find IL-10, PDL1, and PDL2 that could likely be involved in driving the immune-regulatory changes observed during pregnancy in MS patients. These findings suggest that miRNAs may be key players in the induced amelioration of MS symptoms during pregnancy and should be seriously taken into account for future studies [109].

Other relevant evidence shows how MS patients who had elevated ROCK2 and reduced miR-300 and miR-450b-5p levels were linked more pronouncedly in SPMS than in RRMS. Further, ROCK2 and both miRNA levels in RRMS patients with a disability score >5 have not shown differences from SPMS patients. Even though ROCK2 was positively correlated with EDSS, both miRNAs were negatively correlated with it. These evidences show how they could be implied as biomarkers for MS progression [110].

Important evidence is related to hsa-miR-106a-5p as a significant factor in MS pathogenesis, since it was found to be considerably downregulated in the blood of MS patients. Its action has been linked to the regulatory roles it exerts on its targets that are involved in the leukocyte response; thus, its relevant downregulation probably leads to impaired silencing of its target and subsequent dysregulation of the immune response in MS [111].

In this important study, a two-step experiment was assessed using both a bioinformatics approach and an EAE mouse model to validate the data. Indeed, a new set of miRNAs (miR-659-3p, miR-659-5p, miR-684, miR-3607-3p, miR-3607-5p, miR-3682-3p, miR-3682-5p, miR-4647, miR-7188-3p, miR-7188-5p, and miR-7235) has been identified as new possible targets to point out in MS physiopathology due to their ability to aim directly at already important immune-modulatory and inflammation-related genes, such as FXBO33, SGMS-1, ZDHHC-9, GABRA-3, and NRXN-2. The silencing of two of these miRNAs, such as miR-7188-5p and miR-7235, changed the CNS pathophysiology in EAE conditions. These findings highlight the already vast regulatory network existing in MS, giving new targets in lymphoid cells to take into consideration for future therapeutic approaches [112].

miRNA-145 and miRNA-155 expressions are significantly reduced in patients with relapsing-remitting multiple sclerosis (RRMS) compared to healthy controls. These miRNAs have been associated with the regulation of inflammation mechanisms underlying MS; thus, the miRNA-145 and miRNA-155 levels decrease, which may contribute to the pathogenesis of RRMS, and further investigations on both could be useful to better understand their specific roles and mechanisms in MS pathogenesis [113].

Another important investigation is the one conducted on the CSF and serum exosomes of RRMS patients. In this study, it has been found that miRNAs such as Let-7 g-5p, miR-18a-5p, miR-145-5p, and miR-374a-5p, which are known for both pro-inflammatory and anti-inflammatory roles, were significantly upregulated in both CSF and serum-derived exosomes of RRMS patients. On the other hand, miR-150-5p and miR-342-3p, which have anti-inflammatory action, were also significantly upregulated in RRMS patients’ exosomes. Notably, it was also found that anti-inflammatory miR-132-5p and pro-inflammatory miR-320a-5p were significantly downregulated in both CSF and serum-derived exosomes. This peculiar trend might suggest a compensatory anti-inflammatory response in these patients, giving more texture to the intricate mechanism of the ncRNA interactions and functions. As it was already stated before, all these ncRNAs play a real role in their possible future use as biomarkers. The investigation into the differential expression of miRNAs in exosomes derived from CSF and serum could provide valuable insights into RRMS pathophysiology and their future potential as clinical biomarkers [114].

In this work, several miRNAs have been identified in the CSF of PPMS as new possible differential biomarkers to distinguish PPMS from other forms of multiple sclerosis or other neurologic diseases. In particular, let-7b-5p and miR-143-3p were found to be downregulated in PPMS patients CSF; miR-20a-5p and miR-320b were dysregulated in PPMS against RRMS and OND; miR-26a-5p and miR-485-3p were downregulated in PPMS compared to RRMS; and miR-142-5p was upregulated in RRMS compared with other neurological conditions [115].

Balkan and Bilge focused attention on the expression levels of miR-20, miR-21, miR-26, miR-155, and Let-7 miRNAs in RRMS patients and their potential role in the pathogenesis of the disease. Notably, they found that miR-20 was upregulated in the RRMS patients under study, while miR-26 and miR-155 were downregulated. The latter was especially known to act as an important enhancer of pathology in RRMS patients. Its downregulation, along with miR-26, may elucidate new possible roles in the regulatory mechanisms of RRMS patients, potentially affecting disease progression and response to therapy [116].

Other important evidence brought to attention by Shareef et al. highlights the role of the other two miRNAs in the pathogenesis of MS and its clinical worsening. MiR-146a and miR-223 dysregulations, found in individuals with miR-146a rs2910164 and miR-223 rs1044165 genotypes, have a higher risk of developing MS. These findings give new insights not only on the role of ncRNAs as implementable new elements in the MS molecular scenario but also on the genetic variants involved in the population affected by the pathology [117].

In the peculiar and vast context of the miRNA landscape in MS pathogenesis, we must also pay attention to their biogenesis and processing-related genes. Indeed, it was found that genetic variants associated with the AGO1 (rs636832) and GEMIN4 (rs7813) genes are associated with a higher risk of multiple sclerosis, giving more attention not only to the ncRNA interactions and targets but also to the molecular machinery involved in their biogenesis [118].

Given this evidence about the presence of new possible and hopefully future protagonists that could help us enforce the already intricate molecular MS landscape, in silico studies come to help and give more strength to the research on this pathology.

Indeed, in this deep learning study, several miRNAs have been identified to be involved in MS regulatory networks. In particular, hsa-miR-605-5p, hsa-miR-15b-5p, and hsa-miR-16-5p may potentially play significant roles in the pathophysiology of MS. Even though the aforementioned miRNAs have been proposed as molecular actors in MS pathogenesis, their function is not yet detailed, opening new roads for further research and understanding [119].

In this in silico analysis, four important biomarkers have emerged as potential candidates for possible multiple sclerosis treatments. Indeed, their gene targets and associated pathways have shown that mir-142–3p, mir-98–5p, mir-629–5p, and mir-212–3p were found to be statistically significant, and their target pathways and genes, such as PI3K-Akt, MAPK, and JAK-STAT, also mostly converging towards PI3K-Akt, may have an important role in the pathogenesis of MS as well as being new molecular tools in the diagnosis, prognosis, and treatment of MS patients [120].

It has been discovered that genetic variations, such as SNPs, are involved in the regulation of previously known MS-related microRNAs. Using several bioinformatics tools, the researchers have identified the rs540457553 and rs76149940 effects on miR-21 and miR146a/b and how they interact with the attachment of RBP, such as the AGO36 family and DGCR8. Indeed, further research should be focused on the understanding of the genetic landscape underlying the complex network of miRNA regulation [121].

In this study, the main focus is given by the bioinformatics analysis given by the authors, in which they show, for the first time, that hsa-mir-16-5p is the most common involved and likely overlapped in the complex molecular landscape of both AD and multiple sclerosis, addressing new potential roles in the development of new therapeutic strategies and actions toward the pathology. Indeed, it was also found that some hub genes, including CCL2, CD44, GFAP, NEFM, STXBP1, and TCEAL6, are commonly regulated by the aforementioned miRNA [122].

In this study, thanks to literature research, text mining, gene expression analysis, pathway analysis, and genome-wide association studies, the authors have shown that hsa-mir-155-5p, followed by hsa-mir-182-5p, hsa-mir-320a, hsa-mir-148b-3p, and has-mir-301a5p, may serve not only as biomarkers for MS cognitive impairment but also for their important interactions in the complex gene environment underlying MS. Indeed, the most common gene interactions that were found in the analysis have been linked to brain-derived neurotrophic factor (BDNF), which was the most significant gene, followed by interferon-β (IFNB), neurofilament-L (NEFL), neurotrophin-3 (NTF3), and interleukin-10 (IL-10). Other important genes included in the analysis were nerve growth factor (NGF) and chitinase-3-like protein 1 (CH3L1) [123].

Given all these statements about the solid role the miRNAs embrace as biomarkers for MS, key miRNAs such as miR-143.3p, miR-92a.3p, miR-486.5p, miR-142.5p, miR-191-5p, miR-128-3p, miR-24-3p, miR-223-3p, miR-320b, and miR-146a have been reported in our research. These miRNAs are linked to various aspects of the disease, including subtype differentiation, white matter lesion volume, creatinine levels, functional connectivity, and, most importantly, disease progression. This suggests their valuable potential as future biomarkers and also as therapeutic targets in multiple sclerosis (Table 2 and Figure 1).

**Table 2:** The preclinical and human studies that demonstrated alterations in miRNA expression levels could be useful as potential biomarkers in MS.

MS Type	Sample	Experimental Models	MicroRNAs	Role	Ref.
PPMS, RRMS, and SPMS	Blood from patients (serum)	50 MS patients	miR-143.3p ↑↓; miR-92a.3p ↑; miR-486.5p ↑; and miR-142.5p ↓	Potentially useful as biomarkers related to MS severity	[101]
MS	CSF	151 MS patients and EAE mice model	miR-142-3p ↑	IL-1beta/miR-142-3p/GLAST pathway and also as a negative MS biomarker in CSF	[102]
RRMS, SPMS, and PPMS	Blood from patients (serum)	57 MS patients, 18 clinically isolated syndrome patients, and 32 healthy controls over the four-year follow-up	miR-128-3p ↑, miR-191-5p ↑, miR-24-3p nd, and miR-223-3p nd	Potentially useful as biomarkers related to disability accumulation	[103]
RRMS	CSF, serum, and PBMC	10 patients with relapsing MS and 10 HC	↑ miR-15a-3p/124-5p/149-3p/29c-3p/33a-3p/34c-5p/297	Distinctive markers of intrathecal inflammation	[104]
RRMS	Serum	cohort study	↑ miR-320b and miR-25-3p	Used as biomarkers for MS severity	[105]
MS	Serum	30 MS patients and 30 HC	↑ miRNA146a and miRNA155	Higher levels associated with MS disability grade	[106]
MS	Bioinformatics analysis	gene miRNA dataset GSE17846 and mRNA dataset GSE21942	↑ hsa-miR-142-3p, hsa-miR-107, hsa-miR-140-5p, and hsa-mi0R-613	Different target hub genes	[107]
RRMS	Blood samples	32 MS patients and 32 HC	↓ miR-18a-5p	P53 signaling, MAPK signaling pathway, and apoptosis; prognostic biomarkers of high risk	[108]
RRMS	Blood samples	19 pregnant patients	↓ miR-1, miR-20a, miR-28, miR-95, miR-146a, miR-335, and miR-625	Possible makers of the beneficial effects of pregnancy over MS	[109]
SPMS and RRMS	Blood samples	84 Egyptian patients	↓ miR-300 and miR-450b-5p	Changes in the expression levels of ROCK2 and miRNAs 300 and 450b-5p could be useful as biomarkers, as well as related to the degree of disability and MS progression	[110]
MS	Bioinformatics analysis	/	↓ hsa-miR-106a-5p	Possible future biomarkers of MS diagnosis	[111]
MS	Various analysis	EAE mice	↓ miRNA-7188-5p and miR-7235	Downstream inflammatory targets	[112]
RRMS	PBMCs	75 patients and 75 HC	↓ miRNA-145 and miRNA-155	Regulation of inflammatory mechanisms	[113]
RRMS	CSF, serum exosomes	30 untreated RRMS patients and 30 HC	↑ Let-7 g-5p, miR-18a-5p, miR-145-5p, miR-374a-5p, miR-150-5p, and miR-342-3p ↓ miR-132-5p, miR-320a-5p, and miR-17-5p ↑ miR-15a-5p, miR-19b-3p, and miR-432-5p	Possible future use as MS biomarkers	[114]
PPMS	CSF, serum	multicentric study	↓ let-7b-5p; miR-143-3p in CSF; miR-20a-5p and miR-320b, dysregulated in serum; ↑ miR-142-5p in RRMS compared with other neurological conditions	Possible markers in MS to be further studied	[115]
RRMS	Blood samples	20 patients + 20 HC	↑ miR-20, ↓ miR-21, miR-26, miR-155 Let-7	Alterations in the miRNA expression levels in MS patients compared to HC	[116]
RRMS, SPMS, and PPMS	Blood samples	261 patients with MS and 250 HC	hsa-miR-146a and hsa-miR-223	Genotype variants considered as risk factors for MS	[117]
RRMS	Blood samples	194 patients and 188 HC	DROSHA and DICER1 XPO5 RAN AGO1 g variations)	Genotype variants of miRNA processing genes considered as risk factors for MS	[118]
MS	Bioinformatics analysis	deep-learning analysis	hsa-miR-605-5p, hsa-miR-15b-5p, and hsa-miR-16-5p	Could be involved in a possible role in MS pathogenesis	[119]
MS	Bioinformatics analysis	peripheral blood samples from NCBI GEO datasets using GEO2R	mir-142–3p, mir-98–5p, mir-629–5p, and mir-212–3p	PI3K-Akt, MAPK, and JAK-STAT	[120]
MS	Bioinformatics analysis	rs540457553 and rs76149940 SNPs	miR-21 and miR146a/b	Interaction with RNA-binding proteins, such as AGO36 and DGCR8	[121]
MS	GEO database	expression data	hsa-mir-16-5p	Possible new target in MS pathogenesis	[122]
MS	Integrated bioinformatics approaches	integrated bioinformatics approaches	hsa-mir-155-5p, hsa-mir-182-5p, hsa-mir-320a, hsa-mir-148b-3p, and has-mir-301a5p	Interaction with BDNF, IFN-β, NEFL, NTF3, IL-10, NGF, and CH3L1	[123]

MS: multiple sclerosis; PPMS: primary progressive MS; RRMS: relapsing–remitting MS; SPMS: secondary progressive MS; CSF: cerebrospinal fluid; EAE: experimental autoimmune encephalomyelitis; GLAST: glial glutamate-aspartate transporter; HC: healthy controls; PBMCs: peripheral blood mononuclear cells; ROCK2: rho-associated coiled-coil-containing kinase isoform 2; PI3K/AKT: phosphatidylinositol 3-kinase/protein kinase B; JAK-STAT: Janus kinase-signal transducer of activation; BDNF: brain-derived neurotrophic factor; INF-β: interferon-β; NEFL: neurofilament L; NTF3: neurotrophin 3; IL-10: interleukin-10; NGF: nerve growth factor; CH3L1: chitinase-3-like protein 1; ↑: upregulation; ↓: downregulation.

## 5. LncRNAs in Multiple Sclerosis

### 5.1. LncRNAs—Pathway Involved

LncRNAs are crucial factors in MS progression. These types of ncRNAs exert their influence on the disease through their roles in genetic regulation, which affects signaling pathways related to MS [10]. The importance and complexity of the molecular networks that they create have been subjected to many bioinformatics analyses.

For example, Han et al. studied the role of lncRNAs in MS pathogenesis and their interaction with MS-associated SNPs. They identified 2383 brain-specific differentially expressed lncRNAs from 142 individuals (51 MS patients, 91 HC) using RNA-seq data. Of these, 517 lncRNAs were influenced by SNPs. The authors conducted an eQTL analysis, revealing that SNPs remarkably affect the expression of lncRNA and are enriched in neurological diseases and intergenic regions. About 17.6% of lncRNAs in MS showed altered secondary structures due to SNPs. WGCNA and GSEA revealed that SNP regulation of lncRNAs mainly affects antigen processing/presentation and MAPK pathways in MS [124]. The MAPK pathway is an important regulator in the immune-pathogenic mechanisms underlying autoimmune inflammatory diseases of the central nervous system, as evidenced in studies on animal models of MS and in humans [125]. The involvement of the MAPK pathway has also been demonstrated by Sabaie et al., who, through bioinformatics methods, characterized lncRNA-associated ceRNA regulatory networks and the expression profiles in the periplaque regions of MS patients. Further, the team analyzed the expression data from the spinal cord periplaque regions in secondary progressive MS and control samples from the Gene Expression Omnibus (GEO) database. Based on the hypothesis that ceRNA regulatory axes are significantly implicated in MS pathogenesis, they conducted an analysis of these ceRNA axes and the interactions between differentially expressed lncRNAs and mRNAs, leading to the construction of a lncRNA-miRNA-mRNA network. This network comprised three key lncRNAs (TUG1, ASB16-AS1, LINC01094), nine crucial miRNAs (hsa-miR-145-5p, hsa-miR-200a-3p, hsa-miR-20a-5p, hsa-miR-22-3p, hsa-miR-23a-3p, hsa-miR-27a-3p, hsa-miR-29b-3p, hsa-miR-29c-3p, and hsa-miR-34a-5p), and five hub genes (FOS, GJA1, NTRK2, CTNND1, and SP3). Furthermore, the KEGG pathway enrichment analysis revealed significant involvement in the MAPK signaling pathway, Kaposi’s sarcoma-associated herpesvirus infection, human immunodeficiency virus infection, lipids and atherosclerosis, and amphetamine addiction. The findings from this study highlight complex molecular dynamics in the periplaque region, which is an area previously underexplored in the MS landscape, offering potential future therapeutic targets [126].

Moreover, Ghafouri-Fard et al. investigated the expression of MAPK14-related lncRNAs in MS patients and found that NORAD and RAD51-AS1 lncRNAs were upregulated in MS patients compared to controls, while ZNRD1ASP was under-expressed. These expression levels, however, were not correlated with the EDSS score, disease duration, or age at disease onset. This study indicates a dysregulation of MAPK14-related lncRNAs in MS patients, hinting at their possible role in the development of the disease [127].

Through network analysis, Ding et al. investigated the role of ceRNAs in MS. From this research, they identified three pivotal lncRNAs (XIST, OIP5-AS1, and CTB-89H12.4) within the lncRNA-ceRNA network (LACN) linked to MS. Functional analysis revealed that mRNAs adjacent to these lncRNA-ceRNA hubs were enriched in 12 pathways, six of which are directly related to MS, including RNA transport and mTOR signaling. Furthermore, three ceRNA modules centering on XIST, OIP5-AS1, and CTB-89H12.4 were constructed, comprising 96 competitive lncRNA-miRNA-mRNA triplets (LCT). Some of these RNAs were identified as being related to MS. A specific LCT, XIST-miR-326-HNRNPA1, was examined for its expression pattern in MS, indicating a central role in the disease’s pathogenesis. Additionally, they analyzed four miRNAs (miR-125a-5p, miR-140-5p, miR-486-5p, and miR-326), which were associated with MS. For instance, miR-140-5p was significantly reduced in MS patients [128].

Specifically, Safa et al. focused on the role of lncRNAs associated with the NF-κB transcription factor in MS. The NF-κB pathway is a key regulator of immune and inflammatory responses, and it has been shown to be increased in its activation in patients with progressive MS, suggesting an involvement of the latter in the MS pathogenesis processes [129]. They assessed the expression of NF-κB-associated lncRNAs, those related to autophagy, obesity, and two inflammatory cytokines, in the peripheral blood of patients with RRMS and healthy controls. The study aimed to understand the role of lncRNAs in MS and their biomarker potential. A reduced expression of LNC-MKI67IP and HNF1A-AS1, both NF-κB pathway inhibitors, was found in MS patients versus controls, without notable sex-based differences. Contrary to initial hypotheses, the expression of LINC00305 was also found to be reduced in MS patients. This finding was unexpected since LINC00305 is known to enhance inflammatory responses by activating the NF-κB pathway in human monocytes. The study observed increased levels of ADINR and CHAST, suggesting that such correlations may imply an increase in lncRNA levels with age or disease progression. Conversely, no significant differences were found in the expression of MIR31HG, DICER1-AS1, and NKILA between MS patients and healthy controls, despite the fact that these lncRNAs are known to participate in MS-relevant pathways [130].

Instead, Zhang et al. analyzed the role of lncRNA AK018453 and the TRAP1/Smad pathway in the EAE model. They observed increased mRNA and protein levels of TGF-β, TRAP1, TGFβRII, and GFAP in EAE mice compared to controls, with significant upregulation of Smad2/3 phosphorylation. This indicates TRAP1/Smad pathway activation in reactive astrocytes, associated with pro-inflammatory cytokine and chemokine production in EAE mice spinal cords. IL-17 stimulation enhanced TRAP1/Smad pathway activity and pro-inflammatory cytokine production in astrocytes. Increased levels of TRAP1, TGFβRII, GFAP, Smad4, and p-Smad2/3 were observed post-IL-17 stimulation, with augmented TRAP1-Smad4 interaction. This suggests that IL-17 triggers Smad signaling, augmenting pro-inflammatory cytokine production in astrocytes. TRAP1 inhibition in astrocytes significantly reduced TNF-α, CXCL10, and MCP-1 production, indicating an IL-17-mediated TRAP1 upregulation linked to pro-inflammatory cytokine production in astrocytes. TRAP1 knockdown in mice markedly slowed EAE progression, reducing TRAP1, Smad4, p-Smad2/3, GFAP levels, and pro-inflammatory cytokines. Additionally, reduced inflammatory cell infiltration and demyelination in the spinal cord were observed. AK018453 knockdown decreased TRAP1-Smad4 interaction and reduced pro-inflammatory cytokine production in astrocytes. Furthermore, AK018453 knockdown in mice led to decreased EAE progression, reducing pro-inflammatory cytokine production and myelin damage. The results suggest lncRNA AK018453 and the TRAP1/Smad pathway play crucial roles in EAE pathogenesis. They might be a potential therapeutic target for MS treatment [131].

Liu et al. investigated the role of IL-9 and lncRNA Gm13568 in the pathogenesis of MS and its animal model, EAE. IL-9 and Gm13568 impact Notch1 expression and inflammation, with IL-9 raising cytokine production and prompting astrocytes to activate the Notch1 pathway, thereby boosting cytokine levels. The identification of Gm13568 as a lncRNA targeting Notch1 is pivotal. In fact, it regulates the Notch1 pathway and cytokine production in IL-9-activated astrocytes. Gm13568 also interacts with transcriptional co-activators such as CBP/P300 in order to modulate Notch1 expression in astrocytes. Suppressing Notch1 in astrocytes decreases IL-9-induced inflammation. Knockdown of Gm13568 in astrocytes using lentiviral vectors decreases Notch1 pathway activation, significantly reducing inflammation and demyelination in EAE mouse models. These results indicate that Gm13568 regulates cytokine production in active astrocytes, affecting EAE pathogenesis through the Notch1/STAT3 pathway. Gm13568 may serve as a potential target for MS and demyelinating disease treatment [132].

Th17 cells, known for their pro-inflammatory role, are key in MS pathogenesis. These cells are known to produce IL-17, which leads to neuroinflammation and blood-brain barrier breakdown, causing myelin and axon damage in multiple sclerosis. The presence and activity of Th17 cells are particularly noted in MS patients during disease relapses [133,134,135]. Bian et al. investigated the role of the lncRNA Gm15575 in Th17 cell differentiation and the progression of MS and EAE. Overexpression of multiple lncRNAs, notably Gm15575, significantly influenced Th17 cell differentiation, which was demonstrated by alterations in key molecular markers. Notably, silencing Gm15575 reduced the expression of both IL-17A mRNA and protein, as well as RORγt mRNA levels in Th17 cells. Bioinformatics analyses identified Gm15575 as a competing endogenous RNA (ceRNA) that potentially regulates Th17 function through interaction with miR-686. This direct binding between miR-686 and Gm15575 was confirmed through luciferase assays. Further analysis indicated that Gm15575 upregulated CCL7 expression by acting as a ceRNA for miR-686. This interaction indicates the role of Gm15575 in inflammatory cell recruitment and MS exacerbation. Gm15575-miR-686 also controls Th17 cell differentiation and CCL7 secretion. Its silencing in Th17 cells suppressed CCL7 expression, an effect that has been reversed by the suppression of miR-686. These findings disclose how the Gm15575-miR-686 ceRNA network in Th17 cells is indeed important for EAE and MS development [136].

Wang et al. made an integrated analysis to identify key lncRNAs and potential drugs for treating MS. They analyzed gene and miRNA expression profiles from MS patient samples, building a protein–protein interaction (PPI) network and a MS-specific ceRNA network. The authors identified three key lncRNAs (LINC00649, TP73-AS1, and MALAT1) and potential therapeutic drugs through the ceRNA network analysis. They studied the complexity of the interactions within the network, involving lncRNAs, miRNAs, and mRNAs, and how these interactions impact key biological pathways in the MS context, such as the B cell receptor and Epstein–Barr virus infection signaling pathways. The results brought new insights into the pathogenesis of MS and offered a foundation for future investigations into the mechanisms of MS [137].

**Figure 2:**
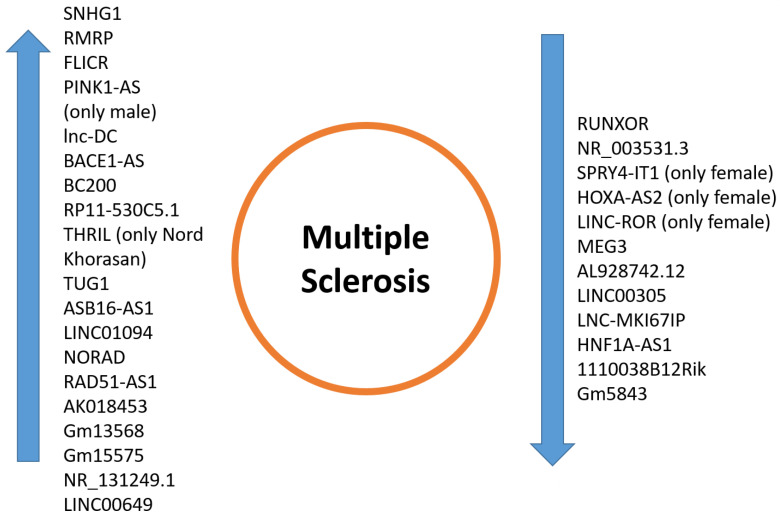
This figure represents the upregulated/downregulated lncRNAs in multiple sclerosis.

These studies support lncRNAs as important factors in MS development, influencing several signaling pathways such as MAPK, mTOR, NF-κB, TRAP1/Smad, and Notch. Data analysis revealed that several lncRNAs are differentially expressed in MS and are influenced by SNPs, altering their secondary structure and affecting pathways linked to antigen processing and MAPK signaling. Other studies have found lncRNA regulatory networks in MS periplaque regions, leading to the creation of a lncRNA-miRNA-mRNA network. These pathways are essential in modulating inflammation and may provide therapeutic targets in MS (Table 3 and Figure 2).

### 5.2. LncRNAs—Biomarkers

LncRNAs might be considered valuable biomarkers for multiple sclerosis, with clinical and pathophysiological implications. Indeed, several studies have highlighted changes in lncRNA expression levels in MS patients, leading to the identification of new useful therapeutic targets and biological markers. Various studies have identified upregulation of specific lncRNAs, such as HIF1A-AS3. Rodríguez-Lorenzo et al. focused on analyzing changes in the choroid plexus (CP) in patients with progressive MS. This lncRNA showed significantly increased expression in patients with progressive MS compared to controls, suggesting a potential role of the disease pathogenesis. Additionally, upregulation of SNHG15 was observed under hypoxic conditions in the CP, indicating its involvement in adaptive responses to hypoxic environments, a condition found in the CP of patients with progressive MS. These alterations in expression may play a significant role in CP function, including neuroprotective factor secretion and immune responses [138].

The small nucleolar RNA host gene (SNHG) family consists of genes encoding small nucleolar RNAs (snoRNAs) within their genomic structures. SnoRNAs are essential in regulating gene expression and RNA maturation [139]. SNHG genes are linked to osteoblast differentiation [140] and are dysregulated in several human diseases, including neonatal pneumonia, diabetic retinopathy, neuropathic pain, acute cerebral infarction, acute myeloid leukemia, endometriosis, glioblastoma, neuroblastoma, and cervical cancer [141].

The study by Sabaie et al. identified variations in an element of this family. Specifically, they elucidated the complex regulatory network of miRNAs and lncRNAs in T cells, focusing on hsa-miR-197-3p and SNHG1, and their impact on the transcriptional regulation of mRNAs in MS. In this context, the interaction between these molecules and their identified targets, YOD1 and ZNF101, could play a significant role in the pathophysiology of MS, offering potential targets for therapeutic intervention [142].

Instead, Dadyar et al. investigated the lncRNAs NEST, RMRP, TH2-LCR, MAFTRR, and FLICR in MS patients and healthy controls, finding significant upregulation of RMRP and FLICR. Strong correlations were observed among these lncRNAs in MS patients, particularly between RMRP and TH2-LCR and between MAFTRR and RMRP, indicating their role in disease pathogenesis. FLICR, which is effective in differentiating MS patients from controls, emerges as a potential biomarker. The expression of NEST positively correlates with MS disease duration and negatively with the age of onset. The involvement of FLICR, a negative regulator of FOXP3 in Tregs, and NEST, which is taking part in activating the IFNG locus, suggests a possible role of these lncRNAs in MS-related immune regulation and inflammation, thus highlighting the importance of T cell-related lncRNAs in MS [143].

The prevalence of MS is significantly higher in women compared to men, with a ratio of about 3:1. However, men may experience a more severe progression of the disease. Sex hormones, estrogens in particular, might influence this gender disparity. Pregnant women often see a reduction in relapses, suggesting a protective effect of hormones. Understanding gender differences in MS, especially in treatment response, still requires more research [144].

Nevertheless, Patoughi et al., analyzing the expression of PINK1 and its antisense non-coding RNA (PINK1-AS) in MS patients versus healthy subjects, found higher PINK1-AS expression in MS patients, particularly in males, suggesting its role in MS pathogenesis. No significant differences in PINK1 expression were observed between MS patients and controls. These results emphasize the potential relevance of PINK1-AS in MS, specifically in male patients [145].

However, the study by Bahrami et al. aimed to investigate the expression of the lnc-DC gene in females with RRMS who were HLA-DRB1*15:01 allele negative, previously associated with the female sex, to understand the role of lnc-DC implied in gender differences in MS pathogenesis. The results demonstrated increased lnc-DC expression in MS patients compared to healthy controls. No significant correlation was found between lnc-DC expression and disease duration, EDSS score, or age of onset. Therefore, the authors propose that the impact of lnc-DC on MS may be more gender-related to these clinical parameters [146].

The attention has also been focused on two important molecular protagonists, such as BACE1-AS and BC200. These lcnRNAs were overexpressed in multiple sclerosis patients. Interestingly, the higher expression of both was observed in MS patients that presented cognitive impairment, while BACE1-AS was higher in remitting cases of RRMS and SPMS groups. The PPMS group had the highest expression of BC200 in both cohorts of MS. It was also found that there is a strong positive correlation between BACE1-AS and BC200, enforcing their role as molecular regulatory actors of MS [147].

On the other hand, there are articles that show lncRNAs are downregulated in MS patients.

The lncRNA RUNXOR, derived from the RUNX1 gene, is implicated in various diseases, such as those related to the immune system. The study by Haridy et al. investigated the role of the RUNXOR/RUNX1 axis in MS development and progression, measuring serum expression levels of RUNXOR, RUNX1, and other critical mRNAs and proteins in MS patients compared to healthy controls. The expression levels of RUNXOR and RUNX1 were significantly reduced in MS patients. This reduction was directly proportional to disease progression, being the lowest in patients with SPMS and the highest in those with clinically isolated syndrome (CIS). Protein levels of RUNX1, NGF, BDNF, MAP2, and IL-10 were also significantly decreased in MS patients, with the reduction intensifying as the disease advanced. RUNXOR, originating from the locus of the transcription factor RUNX1, directly modifies the expression of RUNX1. This interaction plays a pivotal role in the progression of MS. The marked reduction in RUNXOR and RUNX1 levels in SPMS patients corresponds to a lack of neurogenesis, a characteristic of SPMS due to chronic demyelination and axonal deterioration. The study underscores the diagnostic and prognostic potential of the RUNXOR-RUNX1 axis in MS. The significant reduction of RUNXOR and RUNX1 is seen in MS patients, particularly those with SPMS [148].

Instead, the study of Moradi et al. highlights the role of the NR_003531.3 lncRNA as a possible diagnostic biomarker thanks to its direct correlation and detection in MS patients blood samples and not with healthy controls, which strengthens its potential as a future implementable biomarker. The authors demonstrated a down-regulation in the expression of NR_003531.3 in RRMS [149].

Safa et al. observed a significant reduction in the expression of the lncRNAs SPRY4-IT1, HOXA-AS2, LINC-ROR, and MEG3 in female MS patients compared to healthy women. For MEG3, this pattern was also seen in male subjects. Furthermore, HOXA-AS2 expression was correlated with the disease progression index. A significant correlation between HOXA-AS2 and LINC-ROR expression in MS patients was also noted. No other significant correlations between lncRNA expression and clinical data were observed. The correlation between HOXA-AS2 expression and the progression index suggests the potential role of this lncRNA in the latter. Significant associations were found among all lncRNA expression levels in healthy subjects, indicating modifications in the interactive lncRNA network in the MS context. Additionally, sex-based analysis suggested that participant gender might affect this putative network [150].

Ghoveud et al. studied memory B cell lncRNAs near genes with differential expression in RRMS patients and healthy individuals. They found increased expression of lncRNA RP11-530C5.1 in RRMS patients compared to those in remission and healthy controls, indicating its potential role in MS activity as it is upregulated during relapse phases. Conversely, lncRNA AL928742.12 showed decreased expression in RRMS patients, suggesting it may play a role in reducing disease activity. The expression of RP11-530C5.1 and AL928742.12 positively correlated with the PAWR and IGHA2 genes, respectively, hinting at a regulatory interaction with these genes in MS [151].

Instead, Kakhk et al. investigated the expression of the STAT3 gene and its regulatory lncRNAs, lnc-DC and THRIL, in two ethnic groups in Eastern Iran to understand the variances in gene expression among different ethnicities. The case-control study involved MS patients from the Kurdish ethnicity of North Khorasan and the Sistani ethnicity of south-eastern Iran. An increased expression of the THRIL gene was observed in North Khorasan MS patients compared to controls, while Sistani patients exhibited significantly lower expression of both STAT3 and THRIL compared to healthy controls. No significant differences in lnc-DC expression were found. The study highlights that the expression of STAT3 and its regulatory lncRNAs may vary and be differentially regulated in MS patients with diverse genetic backgrounds [152].

These studies have identified specific lncRNAs whose expression changes according to the disease and its clinical phenotypes. Additionally, they highlight how genetic backgrounds, influenced by both gender and ethnicity, demonstrate the complexity of multiple sclerosis pathophysiology. This suggests a need for personalized medicine to effectively address the disease (Table 4 and Figure 2).

## 6. CircRNA inMultiple Sclerosis

Several studies support the hypothesis that circRNAs may serve as biomarkers for multiple sclerosis. For example, Cardamone et al., using RNA-seq on circRNA-enriched samples from PBMCs of 10 MS patients and 10 controls, identified 166 differentially expressed circRNAs in MS patients, with 125 being downregulated. eQTL analysis revealed significant correlations between genetic variants and circRNA expression. A significant association between gene body methylation and circRNA expression was observed, suggesting an epigenetic mechanism in circRNA regulation [153].

Also, Iparraguirre et al. analyzed circRNA expression in MS using RNA-Seq (30 MS patients and 20 healthy controls). They identified 22835 circRNAs with significant global upregulation in MS patients compared to controls. Moreover, 96.1% of differentially expressed circRNAs were upregulated. The study noted sex-dependent expression patterns and proposed six circRNAs as potential biomarkers for MS (PADI4, ABCA13, AFF2, NEIL3, AGFG1, and ATP8B4). This research advances our understanding of circRNA roles in MS and their potential as diagnostic tools [154].

Instead, Zurawska et al. investigated circRNA expression using microarrays and qRT-PCR. They identified 914 differentially expressed circRNAs in RRMS patients during relapses compared to healthy controls. Three specific circRNAs, hsa_circRNA_101348, hsa_circRNA_102611, and hsa_circRNA_104361, were found to have increased expression during relapses and in patients with gadolinium-enhanced brain MRI lesions. Bioinformatics analysis showed 15 miRNAs interacting with circRNAs, affecting B cell function genes [155].

Mycko et al. found significant downregulation of two circRNAs, hsa_circRNA_101145 and hsa_circRNA_001896, in RRMS patients in remission. Ten miRNAs interacting with these circRNAs were identified (for hsa_circRNA_101145: hsa-miR-153-5p, hsa-miR-7-5p, hsa-miR-493-3p, hsa-miR-205-3p, and hsa-miR-181c-5p). For hsa_circRNA_001896: hsa-miR-329-5p, hsa-miR-26a-2-3p, hsa-miR-136-5p, hsa-miR-153-5p, hsa-miR-26a-1-3p) impacting the regulation of genes such as DSE, NOS1, and NOPCHAP1. These results suggest the reduced expression of these circRNAs may play a role in MS progression, offering new avenues for biomarker and therapeutic development [156].

Moreover, Han et al. identified a potential biological process that could link circRNAs and MS. The authors, using microarray analysis and vivo/vitro experimentation, discovered that circINPP4B is a regulator of Th17 differentiation acting on miR-30a as a sponge. This process is important in developing disease. Furthermore, they showed that circINPP4B levels are associated with MS clinical phases; thus, this circRNA could be a diagnostic and therapeutic target. The samples used were 18 RRMS patients and a murine model of EAE [157].

Another interesting article sheds light on the role of one peculiar circRNA, called Circ_0000518. The authors have focused their attention on the possible action it can take in the context of MS. Interestingly, circRNa was found to be upregulated in the peripheral part affected by the disease. circ_0000518 also interacts with FUS protein, which is an RNA-binding protein, which can worsen the disease triggered by the circRNA on its own. It was also found that the interaction triggered by FUS leads to an activation of the CaMKKβ/AMPK pathway and an increased M1 macrophage phenotype, which confirms the pro-inflammatory role of both of these molecular actors [158].

In conclusion, the authors of these articles studied circRNAs in MS contents from a bioinfomatics perspective and the interaction between specific circRNAs and miRNA. The results suggest that circRNAs could be a way of improving MS diagnosis and therapies. therapies (Table 5).

**Figure 3:**
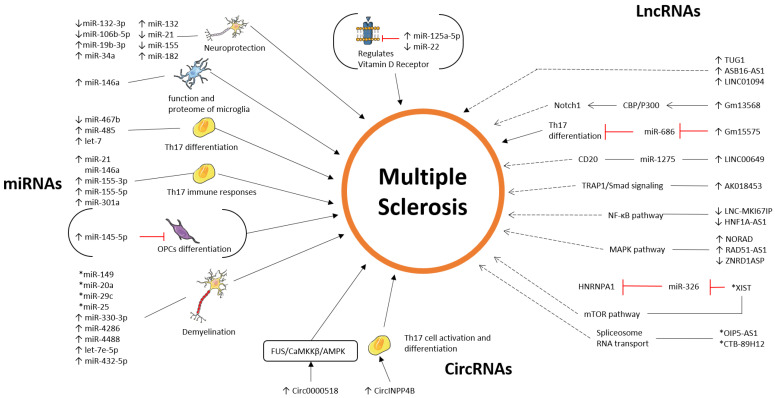
Pathways regulated by lncRNAs, miRNAs, and circRNAs in multiple sclerosis. Black lines represent a direct correlation. The dotted arrows represent an indirect correlation. Normal arrows represent direct interaction. Red lines represent inhibition. ↑: increase; ↓: decrease; *: deregulated.

In the complex molecular landscape of Multiple sclerosis all the ncRNAs that were analyzed cover intriguing functions, depending on their physiological role and their specific cellular type expression (Figure 3).

## 7. Future Perspectives

This review significantly enhances our understanding of MS by exploring the roles of miRNAs, lncRNAs, and circRNAs in disease pathogenesis. It provides a comprehensive overview of the last few years of research regarding how these non-coding RNAs influence MS through their involvement in gene expression regulation, immune function modulation, and their potential as biomarkers and therapeutic targets, while also focusing on some interesting signaling pathways. One of the newest challenges regarding ncRNAs is represented by circRNAs. Even though they are the newest discovered ncRNAs involved in MS pathogenesis [11], they have not been extensively explored, and they further need to be studied in the context of the molecular pathways they are possibly involved in [159]. This issue is not just related to circRNAs; it also involves miRNAs and lcnRNAs. Indeed, to date, there is not yet a comprehensive understanding of their biological role, which becomes a challenge to be explored to use them in the future as diagnostic standards and a possible target of treatments for multiple sclerosis. Furthermore, there is no standardization of sampling methodologies for studying ncRNAs in MS. This review contributes to the current data by offering insights into the molecular mechanisms underlying MS, highlighting the role of ncRNAs in the disease progression, and suggesting new approachable directions for the diagnosis, prognosis, and treatment of this pathology that surely needs to be deeply investigated.

## 8. Conclusions

The ncRNAs, such as miRNAs, lncRNAs, and circRNAs, play roles in the context of MS pathophysiology. The ncRNAs we have covered are particularly involved in Th17 cell activation and differentiation. MiRNAs affect demyelination, Th17 cell activities, vitamin D receptor regulation, and microglia function. lncRNAs impact MAPK, mTOR, TRAP1/Smad, Th17 differentiation, Notch, and NF-kb pathways. circRNAs play roles in Th17 cell physiology and pro-inflammatory pathways like FUS/CaMKKβ/AMPK. Their expression levels are potential biomarkers for MS. Despite the findings highlighted by the available data, there are still few studies on circRNAs; hence, further investigation into their involvement in MS pathophysiology should be assessed.

## Figures and Tables

**Table 1:** The summary of preclinical and human studies that reported the expression level changes of miRNAs and their involvement in pathways related to MS.

MS Type	Sample	Experimental Models	MicroRNAs	Role	Ref.
RRMS SPMS	PBMC and serum	19 MS patients + 14 HC	↓ miR-132-3p, miR-106b-5p, and miR-19b-3p	BDNF increment as a compensatory and protective mechanism	[85]
RRMS SPMS	Blood samples	28 MS patient PBMCs	↑ miR-132-3p, miR-34a, and miR-132	Negative correlation with SIRT1 and BDNF	[86]
RRMS	CSF	25 MS patients and 25 HC	↑ miRNA-21, miRNA-155, and miRNA-182	↑ levels of IL-1β, IL-6, TNF-α, and hs-CRP	[87]
MS	Microglia from mice	miRNA-146a KO mice	miRNA-146a KO	When knocked out, increments of IL-1β, TNF, IL-6, IL-10, CCL3, and CCL2	[88]
RRMS SPMS	PBMCs	40 MS patients and 20 HC	↑ DROSHA in RRMS; ↓ DROSHA in SPMS	↑ IL-6 in SPMS; ↓ of *INF-α* and *INF-β*	[89]
MS	Various analysis	mouse model for EAE	miR-155 role, depending on the cell type	Different roles in infiltrating cells and the MS immune environment	[90]
EAE	Cells	EAE model on mice	↓ miRNA-467b	Targeting of eIF4E suppresses Th17 differentiation, delaying disease progression	[91]
MS		mice	↑ miR-485	↓ STAT3 differentiation of Th17 cells	[92]
MS	Various analysis	mouse model for EAE	↑ let-7	Il1r1 and Il23r, Ccr2, and Ccr5 (it confers protection against EAE-deactivating CD4 cells)	[93]
-	T cell line resembling Th17	C57BL/6J mice and EL4 cell line cells	↑ miR-21, miR-146a, miR-155-3p, miR-155-5p, and miR-301a	T cell gene expression via HSP70 interaction with the RISC complex and miRNAs	[94]
-	OPCs	Rats	↑ mir-145-5p	↓ MYRF prevents OPC differentiation	[95]
SPMS PPMS	/	human brain slices	miR-149*, miR-20a, miR-29c, and miR-25 dysregulation	axonal guidance, TGF-β signaling, and FOXO signaling	[96]
MS	Various analysis	EAE mice	15 miRNAs ↑ 3 miRNAs ↓	KEGG: peroxisome, FoxO signaling, glutathione metabolism, and ferroptosis	[97]
MS	FFPE autopsy tissue	16 autopsied MS patients	↑ miR-330-3p, miR-4286, miR-4488, let-7e-5p, and miR-432-5p	synaptotagmin-7 as a target	[98]
MS	Various analysis	mouse model for EAE	↑ miR-125a-5p	VDR expression ↓	[99]
SPMS RRMS	Serum	50 MS patients and 50 HC	↓ miR-22	↓ VDR and VD levels in MS patients vs. HC	[100]

MS: multiple sclerosis; RRMS: relapsing–remitting MS; SPMS: secondary progressive MS; PBMCs: peripheral blood mononuclear cells; HC: healthy controls; BDNF: brain-derived neurotrophic factor; SIRT1: sirtuin 1; CSF: cerebrospinal fluid; IL-1β: interleukin-1β; IL-6; TNF-α: tumor necrosis factor α; hs-CRP: high-sensitivity C-reactive protein; KO: knockout; interferon-α: INF-α; interferon-β: INF-β; EAE: experimental autoimmune encephalomyelitis; eIF4E: eukaryotic translation initiation factor 4E; Th17: T helper-17; STAT3: transducer and activator of transcription 3; IL1R1: interleukin 1 receptor type 1; Il23r: interleukin 23 receptor; CCR2: chemokine receptor 2; CCR5: chemokine receptor 5; HSP70: heat shock protein 70; OPCs: oligodendrocyte progenitor cells; MYRF: myelin gene regulatory factor; TGF-β: transforming growth factor beta; FFPE: formalin-fixed and paraffin-embedded; VDR: vitamin D receptor; ↑: upregulation; ↓: downregulation.

**Table 3:** The preclinical and human studies showing an alteration in the expression levels of lncRNAs and their involvement in pathways related to MS.

MS Type	Sample	Sperimental Models	LncRNAs	Role	Ref.
Unspecified	blood samples	51 MS patients and 91 controls	2383 differentially expressed lncRNAs	Antigen processing/presentation MAPK pathway	[124]
Unspecified	GSE52139	unspecified	↑ TUG1 ↑ ASB16-AS1 ↑ LINC01094	MAPK pathway Kaposi sarcoma-associated herpesvirus infection Human immunodeficiency virus one infection Lipids and atherosclerosis Amphetamine addiction	[126]
Unspecified	blood samples	12 males and 38 females with MS and 50 controls	↑ NORAD ↑ RAD51-AS1 ↓ ZNRD1ASP	MAPK pathway	[127]
Unspecified	GSE21942, GSE26484, GSE43591, GSE17846, GSE43590, and GSE74579	unspecified	* XIST * OIP5-AS1 * CTB-89H12.4	Spliceosome RNA transport mTOR signaling pathway	[128]
RRMS	blood samples	38 female MS, 12 male MS, 37 female control, and 13 male control	↓ LINC00305 ↓ LNC-MKI67IP ↓ HNF1A-AS1	NF-κB pathway	[130]
-	primary astrocytes	EAE model (C57BL/6 mice)	↑ AK018453	TRAP1/Smad pathway TGF-β/Smad pathway NF-κB p65 CBP/P300 signaling	[131]
-	primary astrocytes	EAE model (C57BL/6 mice)	↑ Gm13568	Notch1/STAT3 pathway	[132]
-	spleens	EAE model (C57BL/6 mice)	↑ Gm15575 ↑ NR_131249.1 ↓ 1110038B12Rik ↓ Gm5843	Th17 cell differentiation	[136]
Unspecified	PBMCs, GSE21942, and GSE61741	12 MS patients and 15 controls	↑ LINC00649 TP73-AS1 MALAT1	B cell receptor signaling pathway and Epstein–Barr virus infection	[137]

MS: multiple sclerosis; lncRNAs: long non-coding RNAs; mTOR: mammalian target of rapamycin; NF-κB: nuclear factor kappa B; TGF-β: transforming growth factor beta; STAT3: signal transducer and activator of transcription 3; Th17: T helper-17; PBMCs: peripheral blood mononuclear cells; *: deregulated; ↑: upregulation; ↓: downregulation.

**Table 4:** The preclinical and human studies that highlight the fact that lncRNAs could potentially be used as biomarkers in MS.

MS Type	Sample	Sperimental Models	LncRNAs	Role	Ref.
Progressive MS	CP samples	6 MS (2 females, 4 males) and 6 controls	↑ HIF1A-AS3 ↑ SNHG15	-	[138]
RRMS	GSE43590 and GSE43591	21 MS patients and 19 controls	↑ SNHG1	-	[142]
RRMS	blood	12 male MS patients, 38 female MS patients, and 50 controls	↑ RMRP ↑ FLICR	-	[143]
RRMS	blood	50 MS patients and 50 controls	↑ PINK1-AS (only male)	-	[145]
RRMS	blood	50 MS female patients and 50 female controls	↑ lnc-DC	-	[146]
RRMS SPMS PPMS	serum	118 MS patients and 20 controls	↑ BACE1-AS ↑ BC200	-	[147]
Clinically isolated syndrome RRMS in relapse RRMS in remission SPMS	serum	120 MS patients and 30 controls	↓ RUNXOR	-	[148]
RRMS	blood	20 MS patients and 10 controls	↓ NR_003531.3	-	[149]
RRMS	blood	40 patients (31 female, 9 male) and 40 controls	↓ SPRY4-IT1 (only female) ↓ HOXA-AS2 (only female) ↓ LINC-ROR (only female) ↓ MEG3 (female and male)	-	[150]
RRMS	PBMCs	50 MS patients and 25 controls	↑ RP11-530C5.1 ↓ AL928742.12	-	[151]
MS	PBMCs	north Khorasan: 30 MS patients (30% male, 70% female) and 30 controls Sistani: 21 MS patients (30% male, 70% female) and 21 controls	↑ THRIL (only north Khorasan)	-	[152]

MS: multiple sclerosis; lncRNAs: long non-coding RNAs; CP: choroid plexus; RRMS: relapsing–remitting MS; SPMS: secondary progressive MS; PPMS: primary progressive MS; PBMCs: peripheral blood mononuclear cells; ↑: upregulation; ↓: downregulation.

**Table 5:** The summary of preclinical and human studies that evaluated changes in the expression levels of circRNAs and their role in biological processes related to MS.

MS Type	Sample	Sperimental Models	circRNAs	Role	Ref.
RRMS	PBMCs	10 MS patients and 10 controls	166 (↓125)	-	[153]
RRMS SPMS	leukocytes	30 MS patients and 20 controls	↑ 22835	-	[154]
RRMS	PBMCs	67 MS patients and 37 controls	914 ↑ hsa_circRNA_101348 ↑ hsa_circRNA_102611 ↑ hsa_circRNA_104361	-	[155]
RRMS	PBMCs	65 MS patients and 37 controls	↓ hsa_circRNA_101145 ↓ hsa_circRNA_001896	-	[156]
RRMS	CD4+ Tn cells	18 MS patients murine model of EAE	↑ circINPP4B	Th17 differentiation	[157]
RRMS PPMS SPMS	cerebrospinal fluid peripheral blood HMC3 cells	56 MS patients and 20 controls murine model of EAE	↑ circ_0000518	FUS/CaMKKβ/AMPK pathway	[158]

MS: multiple sclerosis; circRNAs: circular RNAs; RRMS: relapsing–remitting MS; SPMS: secondary progressive MS; PBMCs: peripheral blood mononuclear cells; EAE: experimental autoimmune encephalomyelitis; Th17: T helper-17; HMC3: human microglial clone 3; FUS: fused in sarcoma; CaMKKβ: Ca^2+^/calmodulin-dependent protein kinase kinase-β; AMPK: AMP-activated protein kinase ↑: upregulation; ↓: downregulation.

## Data Availability

Not applicable.
